# Age-Related Changes in Thymic Central Tolerance

**DOI:** 10.3389/fimmu.2021.676236

**Published:** 2021-04-22

**Authors:** Jayashree Srinivasan, Jessica N. Lancaster, Nandini Singarapu, Laura P. Hale, Lauren I. R. Ehrlich, Ellen R. Richie

**Affiliations:** ^1^ Department of Molecular Biosciences, Institute of Cellular and Molecular Biology, The University of Texas at Austin, Austin, TX, United States; ^2^ Department of Immunology, Mayo Clinic, Scottsdale, AZ, United States; ^3^ Department of Epigenetics and Molecular Carcinogenesis, The University of Texas M.D. Anderson Cancer Center, Smithville, TX, United States; ^4^ Department of Pathology, Duke University School of Medicine, Durham, NC, United States; ^5^ Livestrong Cancer Institutes, Dell Medical School, The University of Texas at Austin, Austin, TX, United States

**Keywords:** central tolerance, life span, thymus, thymic epithelial cells, dendritic cells

## Abstract

Thymic epithelial cells (TECs) and hematopoietic antigen presenting cells (HAPCs) in the thymus microenvironment provide essential signals to self-reactive thymocytes that induce either negative selection or generation of regulatory T cells (Treg), both of which are required to establish and maintain central tolerance throughout life. HAPCs and TECs are comprised of multiple subsets that play distinct and overlapping roles in central tolerance. Changes that occur in the composition and function of TEC and HAPC subsets across the lifespan have potential consequences for central tolerance. In keeping with this possibility, there are age-associated changes in the cellular composition and function of T cells and Treg. This review summarizes changes in T cell and Treg function during the perinatal to adult transition and in the course of normal aging, and relates these changes to age-associated alterations in thymic HAPC and TEC subsets.

## Introduction

**Figure 1:**
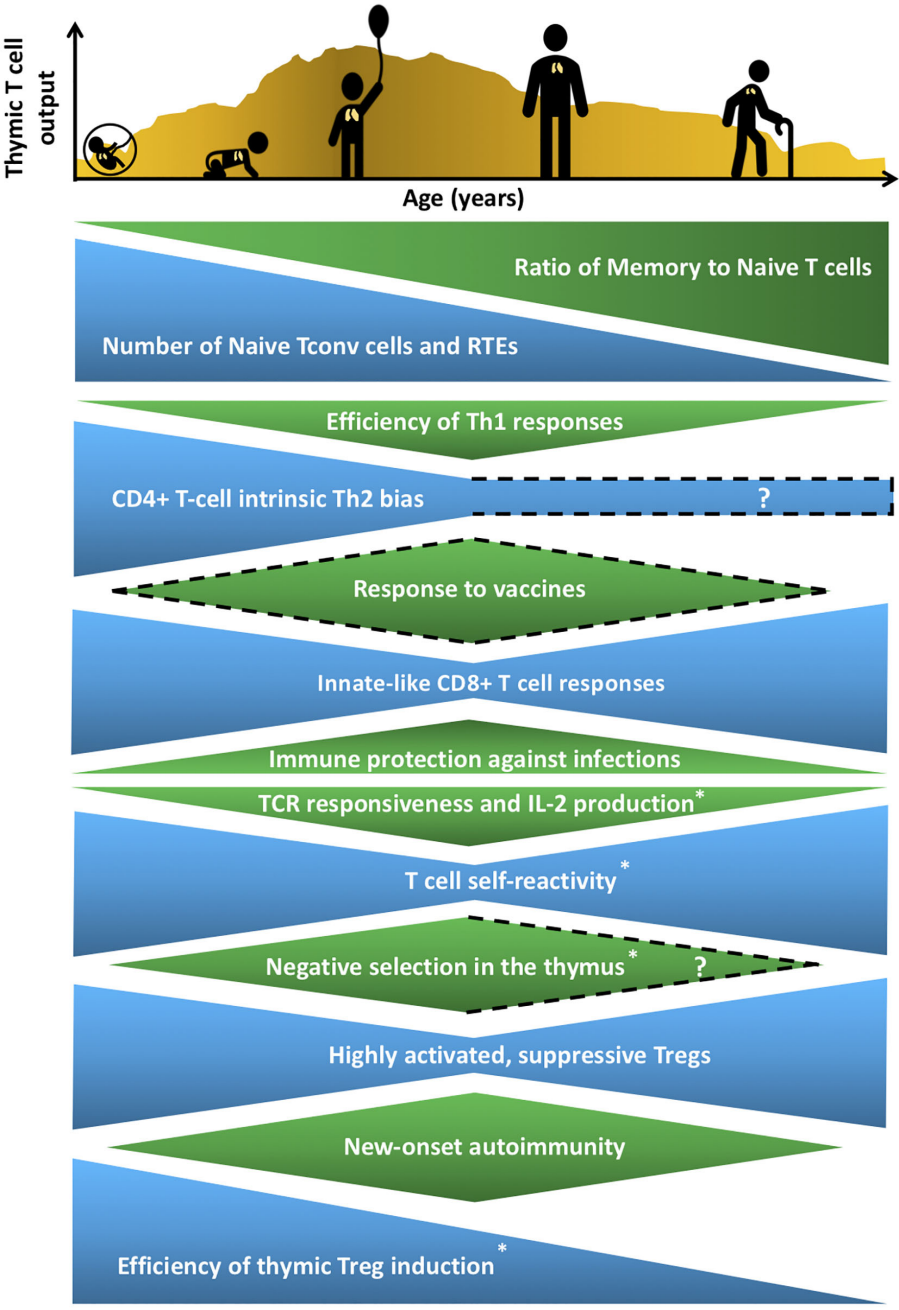
Age-associated changes in T cell generation and function throughout the lifespan. The T cell landscape is in flux throughout life, shaped by age-associated changes in T-cell subset composition and function, which are influenced by cell-intrinsic factors as well as microenvironmental cues that support T cell development and differentiation. While the perinatal T cell pool is dominated by naive conventional T cells (Tconv) and recent thymic emigrants (RTEs), the aged T cell pool contains a higher proportion of memory T cells. Perinatal and aged T cells share several striking similarities in phenotypes and functions. The perinatal and aged CD8+ Tconv cells, including virtual memory T cells (Tvm), are shifted towards short-lived, innate-like, effector responses characterized by increased proliferative potential and rapid cytokine production, at the expense of long-lasting memory generation. Naive CD4+ T cells also display age-associated changes at both ends of the age spectrum, such as reduced T cell receptor (TCR) responsiveness and IL-2 production. In addition, T cells are more self-reactive both early and late in life, which may reflect age-associated changes in thymic selection and/or peripheral maintenance. Regulatory T cells (Treg) generation in the thymus peaks in the perinatal period, but Tregs at both ends of the age spectrum have superior suppressive capacity compared to adult Tregs. These age-associated changes implicate the thymic microenvironment in selecting Tconv cells and Tregs that cater to rapidly changing immune challenges throughout life, while at the same time curbing the risk of triggering autoimmunity. T cell output from the thymus is also lower in both fetal/neonatal periods as well as in the elderly. The uneven pattern of thymic output depicted in the histogram reflects variability throughout life due to numerous extrinsic stressors, such as infections and pregnancy, that alter thymic cellularity and output. In keeping with the above similarities between T cells in the perinatal and elderly stages, immune outcomes, such as overall responsiveness to vaccines and pathogens, as well susceptibility to new onset autoimmunity change in similar directions at both extremes of the lifespan. Phenotypes with question marks are yet to be defined clearly, and dotted lines indicate variable findings in the indicated attributes. All features have been reported in both humans and mice, except those denoted with an asterisk that indicates findings currently reported only in mice in the perinatal to adult and/or adult to aged transitions.

Throughout life, the immune system must balance the opposing goals of mounting protective responses against diverse pathogens, while preventing a breakdown in self-tolerance. Maintaining this tenuous balance is complicated by age-related changes in the number and composition of cells that comprise the innate and adaptive immune systems, as well as by changes in hematopoiesis, lymphoid and non-lymphoid tissue microenvironments, and an individual’s history of pathogen exposure. Neonates encounter a barrage of new pathogens, requiring broad and rapid immune protection, at a time when their immune system is skewed towards mounting tolerogenic responses essential for tissue homeostasis (1). In contrast, following a lifetime of pathogen encounters, the T-cell compartment in older individuals contains a higher frequency of memory T cells, which can combat previously encountered pathogens, but often mounts poor responses to newly encountered pathogens and vaccines (2). (**Figure 1**). Immune responses to self-antigens also exhibit age-associated trends with the onset of many autoimmune disorders peaking in middle age (3) (**Figure 1**). Notably, there are some similarities between manifestations of immune dysregulation at both ends of the age spectrum, as neonates and elderly individuals have elevated susceptibility to various pathogens relative to adults, but less susceptibility to new-onset autoimmune disorders. For example, neonates are highly susceptible to respiratory syncytial virus (RSV) (4), whereas elderly individuals often mount inadequate immune responses to influenza and West Nile viruses (5, 6). T cells play a central role in modulating the outcome of immune responses by integrating initial signals from the innate immune system with T cell receptor (TCR)-mediated antigen recognition to shift the balance in favor of pathogen-directed protective versus tolerogenic outcomes. Distinct T-cell subset composition, phenotypes, and effector functions have been identified in neonates and in aged individuals compared to younger adults, but the underlying mechanisms responsible for the distinctive age-associated features of T-cell immunity have not been fully established.

**Figure 2:**
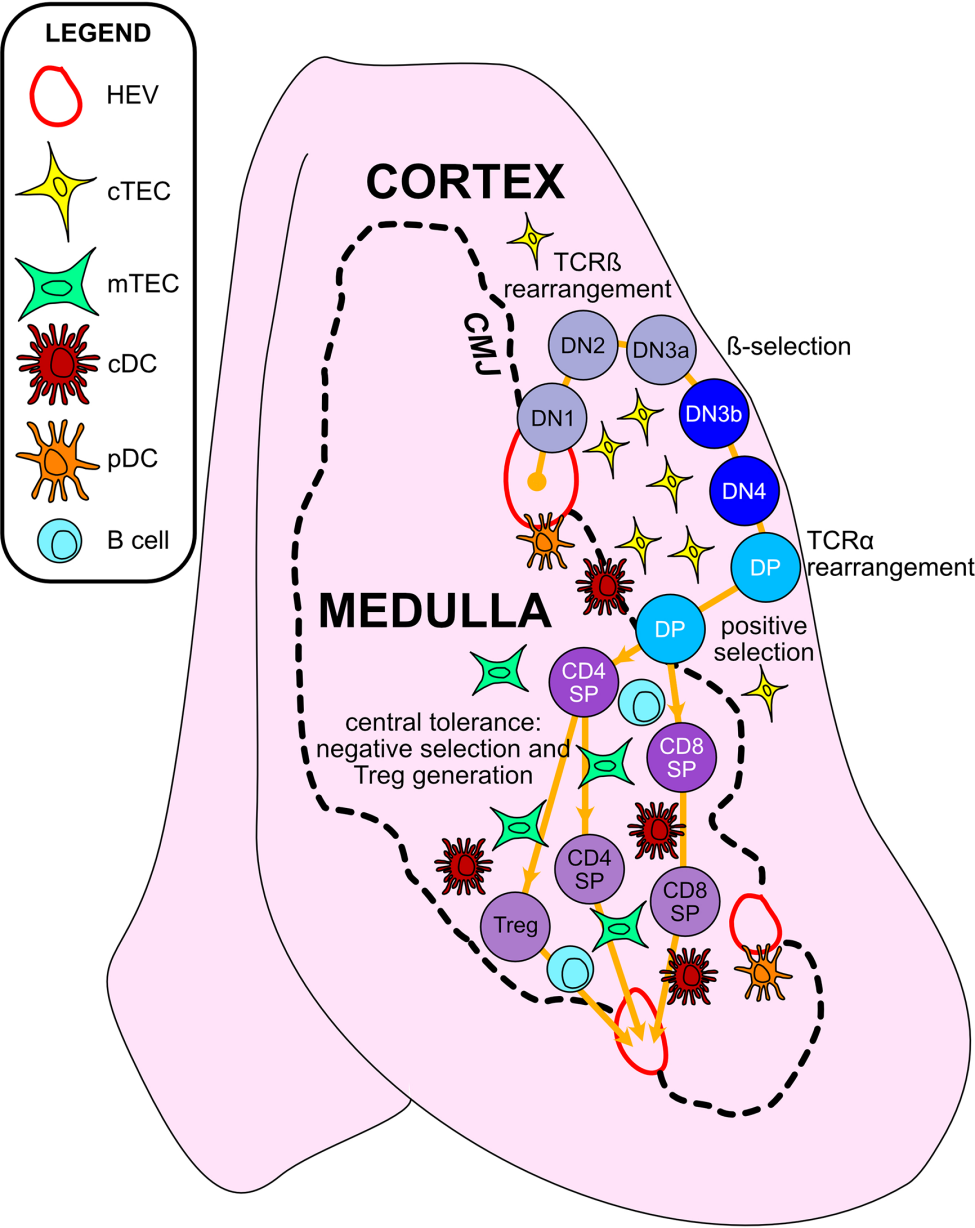
Thymic epithelial cells and hematopoietic antigen presenting cells provide essential signals to guide αβT cell maturation and the induction of central tolerance in the thymus. Cross-sectional view of a thymus lobe reveals cortical and medullary regions, through which thymocytes must travel in an orchestrated manner to encounter heterogeneous stromal cell subsets. Progenitor cells from the bone marrow migrate through the vasculature to seed the thymus at the cortico-medullary junction (CMJ). DN1-DN4 thymocytes require signals from cortical thymic epithelial cells (cTECs) to support their survival, proliferation, and T-lineage commitment. During the DN2-DN3 stages, TCRβ gene segments are recombined, and thymocytes that successfully express TCRβ and signal through the pre-TCR undergo proliferation and further differentiation through the process of β-selection. Subsequently, thymocytes upregulate CD4 and CD8 to become double-positive cells (DPs), which initiate TCRα gene rearrangements. DPs that successfully express a TCRαβ heterodimer are tested for reactivity with self-peptide MHC complexes presented by cTECs. Only those DPs that receive a TCR signal pass positive selection, enabling them to survive and further differentiate. Positively selected DPs transit from the cortex into the medulla. Along the way, some clones may be deleted in an early wave of negative selection in the cortex, driven by strong TCR reactivity to self-peptide MHC complexes displayed by dendritic cells (DCs). In the medulla, DPs downregulate either CD4 or CD8 to become single-positive thymocytes (CD8SP or CD4SP) and interact with medullary APCs to establish central tolerance to a broad array of self-antigens. Strong TCR signals, induced by self-antigens displayed by medullary thymic epithelial cells (mTECs), conventional DCs (cDCs), plasmacytoid DCs (pDCs), or B cells result in either negative selection (apoptosis) or Treg diversion of the autoreactive T cell clones, enforcing central tolerance. SPs that survive these collective thymic selection processes emigrate from the thymus to join the peripheral T cell pool.

T cells develop in the unique tissue microenvironment of the thymus (**Figure 2**), in which thymic epithelial cells (TECs) and hematopoietic antigen presenting cells (HAPCs) provide indispensable signals for T-cell maturation and/or the establishment of self-tolerance. Bone-marrow derived hematopoietic progenitors are recruited from circulation into the postnatal thymus. These CD4^-^CD8^-^ “double negative” (DN) precursors then undergo T-cell lineage specification and differentiation in the thymic cortex. Following productive rearrangement of TCRβ gene segments, DN thymocytes initiate expression of the TCR co-receptors CD4 and CD8 and are referred to as “double positive” (DP) cells. DPs undergo TCRα gene rearrangements resulting in expression of functional αβTCR heterodimers that scan self-peptide/MHC complexes (pMHC) presented by cortical thymic epithelial cells (cTECs). Only thymocytes that express a TCR of sufficient affinity for either MHCI- or MHCII-peptide complexes are signaled to survive and further differentiate to CD8^+^ or CD4^+^ single positive (SP) lineages, respectively, through the process of positive selection (7, 8). A range of TCR affinities is compatible with positive selection, and the level of thymocyte self-reactivity has been shown to affect the subsequent threshold of peripheral T cell activation. Positively selected thymocytes migrate into the medulla, a region specialized for the induction of central tolerance. Within the medulla, TECs and HAPCs display a diverse array of self-peptides. Thymocytes expressing TCRs of relatively high affinity for self-pMHC are either triggered to undergo apoptosis, through the process of negative selection, or are diverted to a regulatory T cell (Treg) lineage to establish central tolerance (9). The combined outcomes of positive selection and central tolerance shape the specificity, diversity, and self-reactivity of the TCR repertoire in the peripheral T cell compartment.

**Figure 3:**
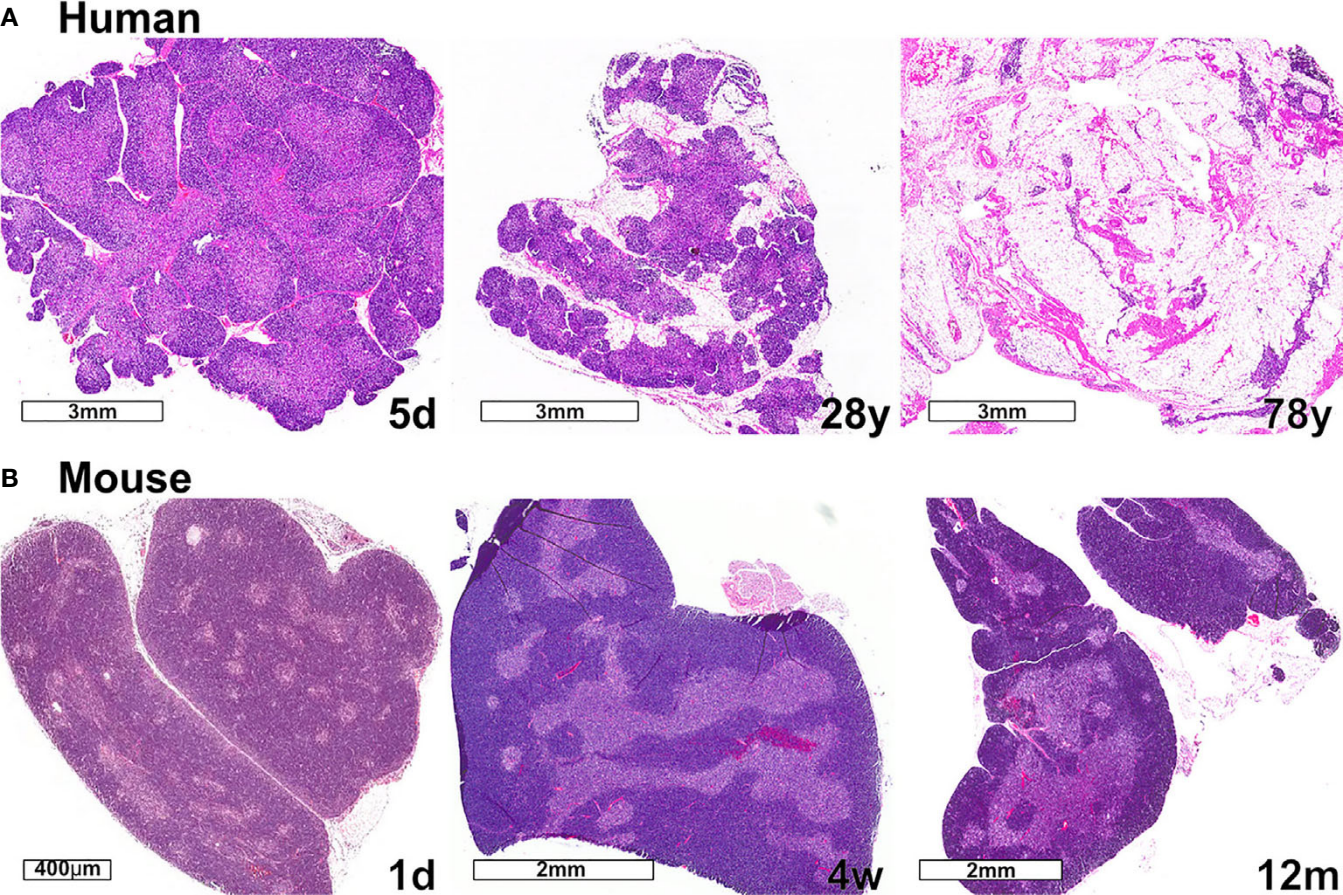
Changes in thymic size, organization, and/or lipid content accompany age-associated thymic involution in humans and mice. **(A)** In humans, the percentage of the thymus comprised of functional thymic tissue progressively declines with age, and is replaced by adipose tissue, as shown in these hematoxylin and eosin-stained images. The percent of thymus area containing thymic epithelium, representing functional thymic tissue, was calculated *via* morphometric analysis of cytokeratin immunohistochemical slides. The results for the subjects shown are 91% at 5 days (5d), 55% at 28 years (28y), and 0.5% at 78 years (78y). **(B)** The mouse thymus grows substantially between postnatal day 1 (1d) (scale bar = 400 µm) and 4 weeks of age (4w) (scale bar = 2 mm), and then declines steadily and is highly involuted by 12 months of age (12m). The small islands of medullary tissue seen at 1d expand and coalesce to form the larger, more organized medullary regions characteristic of adult thymus (4w). Age-associated replacement by adipose tissue is not a prominent characteristic of involution in mice. The corresponding weights (mean ± SD) of murine thymus at the ages shown are 5 ± 0.5 mg at 1d (n = 3), 57 ± 8 mg at 4w (n = 8), and 38 ± 2 mg at 12 m (n = 3).

Changes in thymus size and thymocyte cellularity are the most apparent age-related changes in the thymus. In both humans and mice, thymus size continues to increase in the neonatal period, then transitions to a homeostatic phase during early life, prior to the initiation of progressive age-associated involution. While the age-associated decline in size and output of T cells is conserved between mice and humans, one notable difference is that only human thymuses accumulate high levels of lipid laden adipocytes, which are interspersed with relatively small functional regions of thymic tissue (**Figure 3**). Accumulating evidence discussed below indicates that the cellularity and composition of TECs and thymic HAPCs change with age. As TECs and thymic HAPCs play critical roles in establishing central tolerance, age-related changes in the thymic microenvironment likely impact thymocyte selection, tolerance, and thus peripheral T cell responses throughout the lifespan. In this review, we focus on age-associated changes in the thymic microenvironment that can affect the diversity and self-reactivity of T cells that emigrate into the periphery to participate in immune responses. We first review the establishment of central tolerance and the roles of TECs and HAPCs in this process. We then discuss age-associated characteristics of conventional and regulatory T cell responses and how they may be linked to changes in thymic selection. Finally, we explore age-associated changes in the composition of HAPC and TEC subsets that may contribute to altered central tolerance and T cell activity throughout life.

## The Players in Thymic Central Tolerance

TCR gene rearrangements can generate >10^15^ distinct TCRs, enabling recognition of an extensive array of diverse antigens (10, 11). Given the random nature of the TCR gene rearrangement process, it is inevitable that some TCRs will recognize self-antigens. To achieve self-tolerance, thymocytes must be screened for autoreactivity and either purged or directed into the Treg lineage to prevent autoimmunity. Multiple factors influence whether a self-reactive thymocyte will undergo negative selection or Treg lineage diversion. One critical determinant is the avidity of TCR binding to pMHC complexes presented by thymic APCs, which is a combined function of both individual TCR-pMHC binding affinities and the abundance of pMHC on APC surfaces. High-avidity binding results in thymocyte negative selection, eliminating autoreactive clones from the TCR repertoire (7). Selection into the Treg lineage is generally induced by somewhat lower avidity TCR-pMHC interactions (9). However, the broad and partially overlapping TCR repertoires of conventional T cells (Tconv) and Tregs (12) demonstrate that this cell fate decision is not dictated solely by TCR avidity. Another factor influencing fate choice is intraclonal competition for limited Treg niches. Thymocytes expressing a Treg-derived TCR transgene efficiently divert to the Treg lineage only when present at low clonal frequencies (9, 13, 14). Thus, the fate of a self-reactive thymocyte is determined by cell-intrinsic and -extrinsic factors. Cell-extrinsic factors include the abundance and local availability of self-pMHC (15–17), CD80 and CD86 co-stimulatory molecules (18, 19), and IL-2, with some contribution from IL-15 and IL-7 (20, 21). Altogether, multiple factors in the thymic environment shape the self-reactivity and diversity of emerging T cells, regulating their responsiveness to self- and foreign antigens.

A variety of thymic APC types present self-peptides to induce central tolerance. The importance of the thymic medulla in negative selection is well-established. Nevertheless, two studies reported that most antigen-induced clonal deletion occurs in DP thymocytes, suggesting that cortical APCs can induce negative selection (22, 23). Although antigen presentation by cTECs is required for positive selection, cTECs have not been associated with negative selection (7). Instead, several studies indicate that HAPCs, such as DCs, in the cortex and near the cortico-medullary junction (CMJ) present ubiquitous self-antigens to induce cortical negative selection (24–26). Thymocytes clearly undergo negative selection at the DP stage, but further studies are needed to determine if DPs undergo negative selection in the cortex or medulla. Prior research predominantly relied on CCR7 expression as a proxy for cortical versus medullary localization of thymocytes undergoing negative section. However, live imaging studies indicate that positively-selected DPs can enter the medulla before upregulating CCR7 (27, 28), raising the possibility that medullary APCs may also contribute to this early wave of DP negative selection.

Thymocytes are screened for reactivity against non-ubiquitous self-antigens primarily in the medulla. The requirement for medullary localization was demonstrated in mice deficient for the chemokine receptor CCR7 for its ligand CCL21, which together promote the medullary accumulation of post-positive selection SP thymocytes (29–33). In the absence of CCR7 signaling, thymocyte migration into the medulla is compromised, resulting in diminished central tolerance and subsequent autoimmune exocrinopathy (34). mTECs play a key role in negative selection due to their unique ability to collectively express >85% of the proteome, allowing them to induce central tolerance against a wide array of self-antigens (35–37). Importantly, mTECs express tissue-restricted antigens (TRAs), encoded by 2,000-3,000 genes that are otherwise expressed only in a small number of terminally-differentiated tissues (38–40). TRA expression is largely under control of the transcriptional regulator AIRE (35–37), which is expressed predominantly by MHCII^hi^ CD80^hi^ mature mTECs (41, 42). AIRE deficiency impairs mTEC maturation and prevents expression of Aire-dependent TRAs, resulting in failed central tolerance and export of autoreactive T cells that induce multi-organ autoimmunity (43, 44). Analogous to *Aire*-deficient mice, autoimmune polyendocrinopathy-candidiasis-ectodermal dystrophy (APECED) patients have mutations in the *AIRE* gene, resulting in autoimmunity affecting multiple endocrine glands (45, 46). TCR repertoire analysis of Treg and Tconv cells from *Aire*-deficient and -sufficient mice demonstrated that AIRE is also required to select autoreactive clones into the Treg lineage (47, 48).

In addition to expressing diverse self-antigens, mTECs play a critical role in central tolerance by directly presenting self-antigens to thymocytes. MHCII^hi^ mTECs express the costimulatory molecules CD80 and CD86, which are required for negative selection and Treg induction (49, 50). Reducing MHCII expression selectively in mTECs revealed that efficient negative selection requires antigen presentation by mTECs (51). Moreover, tolerance to the *Aire*-dependent RIP-mOVA neoantigen remained intact when MHC was expressed only by TECs, and not by thymic HAPCs (52, 53). Furthermore, our imaging studies using thymic slices from RIP-mOVA transgenic mice, in which MHC is physiologically expressed by both mTECs and HAPCs, revealed that AIRE+ mTECs contribute to roughly half of the negatively-selecting interactions with both MHCI and MHCII-restricted thymocytes (54). mTECs also have the capacity to induce Tregs independently of HAPCs (55). Interestingly, while AIRE^+^MHCII^hi^ mTECs clearly express and present diverse TRAs that are essential for central tolerance, recent findings demonstrate that MHCII^lo^ mTECs also contribute to selection of the T cell repertoire (56). Thus, mTECs play a pivotal role in central tolerance, not only as sources of TRAs, but also as APCs that directly present self-antigens to thymocytes to induce central tolerance.

The importance of HAPCs, in particular thymic dendritic cells (DCs), in negative selection is well-established, as genetic ablation of DCs leads to defective central tolerance and autoimmunity (57). DCs efficiently mediate negative selection and Treg induction in reaggregate thymic organ cultures (RTOCs) (58). *In vivo*, DCs can acquire antigens in peripheral tissues and traffic them into the thymus to induce tolerance (59), and those positioned near thymus vasculature can acquire and present blood-borne antigens (60, 61). Thymic DCs also acquire and present mTEC-derived TRAs (53, 62, 63). Thymic DCs with an activated signature, including elevated expression of MHCII and CD86, function as highly efficient APCs (64). Thymocyte-DC crosstalk is important for DC maturation and function. CD40L on SP thymocytes induces CD40 signaling in DCs, which is required for DCs to induce Tregs *in vitro* (65). Interestingly, TCR repertoire sequencing demonstrated that mTECs and DCs select distinct clones into the Tconv and Treg repertoires (48, 66). Our imaging studies also indicated that DCs present mTEC-derived TRAs to MHCI- and MHCII-restricted thymocytes (54). For both monoclonal TCR transgenic and polyclonal thymocytes, DCs were engaged in slightly more than half of the interactions between thymocytes and APCs that induced TCR signaling (54), further supporting the fundamental contribution of DCs to central tolerance. Collectively, these studies demonstrate that both mTECs and DCs are required to establish self-tolerance.

DCs are a heterogenous group of HAPCs that include conventional DCs (cDC) and plasmacytoid DCs (pDCs) (67). The mouse cDC1 subset expresses CD8α and XCR1, and the cDC2 subset expresses CD11b and Sirpα/CD172a (68). While both cDC subsets contribute to tolerance induction, thymic cDC2s have greater CD4+ T cell stimulatory capacity (69) and are especially proficient at Treg induction (70, 71). The cDC1 subset plays a role in clonal deletion, but was reported to be dispensable for Treg induction and to have a negligible impact on the Treg TCR repertoire (72, 73). In contrast, other studies concluded that cDC1s are essential for inducing Tregs in response to mTEC-derived antigens (48, 66). Thus, further studies are needed to resolve the contributions of distinct DC subsets to central tolerance. When compared to cDCs, pDCs in the thymus have a reduced capacity to stimulate T cells and to acquire antigens from TECs (74). However, CCR9+ pDCs can transport peripheral antigens to the thymus to induce negative selection (75). In humans, comparable cDC1 and cDC2 subsets have been identified and defined by expression of CD141 and CD1c, respectively (68). It has been technically challenging to dissect the roles of human thymic APC subsets in establishing central tolerance, although *in vitro* studies have confirmed that DCs have tolerogenic capacity (76), including the ability to induce Tregs (77). Human thymic cDCs are activated by thymic stromal lymphopoietin (TSLP), which is expressed by Hassall’s corpuscles that consist of terminally differentiated mTECs, and the activated CD80^hi^ CD86^hi^ cDCs can induce Tregs *in vitro* (78). Like cDCs, human thymic CD123+ pDCs support Treg induction *in vitro* (79, 80). Thus, multiple thymic DC subsets have been shown to promote central tolerance, though the distinct contributions of DC subsets are not entirely resolved.

B cells have also been shown to contribute to central tolerance. Thymic B cells are localized in the medulla and express high levels of MHCII, CD80, and CD86, distinguishing them from splenic B cells (81, 82). Thymic B cells with specificity for self-antigens can present self-peptides to CD4SPs, driving activation-induced cytidine deaminase (AID)-dependent B cell class switching. Class-switched thymic B cells promote negative selection (81, 82). CD40 activation in thymic B cells, driven by CD40L on SP thymocytes, is required to support B cell proliferation, differentiation, and class switching, as well as upregulation of *Aire*, and these licensed B cells present self-antigens to induce negative selection (83, 84). Thus, B cells may play a significant role in central tolerance, but whether licensed B cells are autoreactive and the nature of the self-antigens they present remain to be resolved. Collectively, these studies demonstrate the cooperative roles of multiple TEC and HAPC subsets in enforcing central tolerance against a broad range of self-antigens.

## Changes in Tconv Function and Thymic Selection Throughout the Lifespan

### Function of Tconv Cells in the Perinatal Period

Tconv cells generated during the perinatal period face the daunting task of mounting a rapid, protective immune response against a sudden surge of pathogen encounters, while at the same time ensuring they do not trigger autoimmunity. Neonatal T cells differ substantially from adult T cells in composition and function, helping them to achieve this balance (85). Generally, T cell responses in neonates are diminished relative to those of adults. This may partly be attributed to a shift in the ratio of naive to memory subsets, as naive T cells are predominant in perinatal tissues, whereas memory T cells become more abundant in adults (86). In keeping with this concept, T cells from pediatric lymph nodes (LNs) produce relatively lower levels of cytokines, including IFN-*γ*, IL-2, and IL-4 relative to adult T cells (86). Moreover, in the context of infections such as malaria (87) and congenital Cytomegalovirus (CMV) (88) human neonatal T cells express lower levels of Th1 and Th2-associated cytokines compared to adult T cells. Cell-intrinsic properties of perinatal T cells such as high PD-1 expression (88), low NFAT expression (89) and diminished Ca^2+^ influx after TCR stimulation (90) may contribute to the diminished responsiveness of neonatal T cells. Overall, the relative paucity of memory T cells and reduced functionality of T cells in the perinate are consistent with increased susceptibility to infection in early life.

Multiple studies in mice and humans have demonstrated that neonatal T cell responses are strongly skewed towards Th2 versus Th1 differentiation (91–95). Mouse neonatal T cells and human cord blood T cells readily produce the Th2 cytokines IL-4 and IL-13 following *in vitro* stimulation (92, 94–96). Also, CD4+ T cells isolated from neonatal mice immunized with bacille Calmette-Guerin (BCG), Tetanus toxoid and other vaccines expressed higher levels of IL-5 and lower IFN-*γ* upon antigen re-stimulation *in vitro*, compared to adults (97). The neonatal Th2 bias is due at least in part to permissive epigenetic regulation of Th2-associated cytokine genes (95, 98). Also, the IFN-*γ* promoter region is hypermethylated in cord blood CD4+ T cells consistent with their deficient production of IFN-*γ*  after *in vitro* stimulation (99). Moreover, stimulated CD4+ cord blood T cells express higher levels of GATA3, a key transcriptional regulator of Th2 fate (94, 100). The strong Th2 bias could be beneficial both in suppressing development of damaging inflammatory Th1 responses, as well as in promoting tolerance towards allogeneic maternal antigens *in utero*. Consistent with the latter idea, cord blood from preterm infants contains higher levels of proinflammatory cytokines and alloreactive Th1-like central memory CD4+ T cells, which were absent in term infants, suggesting their potential role in promoting premature uterine contractions (101). However, Th2-skewing could leave the newborn vulnerable to infections and unable to respond to some vaccines, which require Th1 responses (102–104).

Interestingly, studies have demonstrated that with appropriate stimuli, such as exogenous IFN-*γ* and IL-12 (105, 106), exposure to helminth and mycobacterial antigens (107), low viral doses (108), various adjuvants (93), or DNA vaccines (109), neonates can mount Th1-like responses in addition to Th2 responses (110–113). In contrast to findings in mice (97), BCG vaccination of infants induces a strong Th1 response, comparable to adults, supported by high IFN-*γ* and low IL-4/IL-5 expression after antigen re-stimulation *in vitro* (112, 113). Moreover, Th1 responses are elicited by CMV in the fetus and *B. pertussis* in infants (88, 114). The capacity of neonatal T cells to mount a Th1 response under some conditions may reflect the extent of DC maturation, as mycobacterial and pertussis toxin antigens are particularly effective at activating DCs (115, 116). Nonetheless, studies with neonatally immunized mice suggest that while Th1 responses can be induced in adults following antigen re-challenge, Th2 memory responses still predominated (93, 117). In addition, while a balanced Th1 and Th2 primary response could be induced in neonates early after exposure to a foreign antigen, a Th2 secondary response was dominant in mice re-challenged as adults (111).

Cell-intrinsic properties of neonatal T cells, as well as extrinsic microenvironmental cues have been implicated in driving the reduced responsiveness and Th2 bias of neonatal T cell responses. Adoptive transfer experiments in mice revealed that Th2 skewing was observed only when fetal, but not adult CD4+ T cells were primed regardless of whether the host microenvironment was fetal or adult (110, 118, 119). These results suggest a cell-intrinsic difference in the fate potential of neonatal versus adult CD4+ T cells. Interestingly, when both Th1 and Th2 responses were elicited by primary antigen challenge in neonates, Th1 cells upregulated IL-13Rα1 which associated with IL-4Rα (119). Upon antigen re-challenge, the activated Th2 cells secreted IL-4 which bound the IL-4Rα/IL-13Rα1 heterodimer, triggering Th1 apoptosis, tipping the balance towards Th2-mediated immunity. Moreover, upregulation of IL-13Rα expression during initial activation of Th1 cells is developmentally regulated; antigen exposure after postnatal day 6 does not induce IL-13Rα expression. These results are due to the delayed maturation of a subset of splenic CD8α+ cDC1s, which secrete IL-12 that inhibits IL-13Rα expression on Th1 cells (120). These findings demonstrate that cell extrinsic factors can regulate the Th2 bias in neonates.

Neonatal CD8+ T cell responses also differ from their adult counterparts (reviewed in (85)). Co-transfer of neonatal and adult CD8+ T cells into adult recipients revealed a cell-intrinsic bias of neonatal cells towards a short-lived effector fate, whereas adult T cells differentiated into both effector and memory subsets (121). Thus, upon pathogen re-challenge, the immune response was dominated by adult CD8+ T cells. Further studies demonstrated that neonatal and adult CD8+ T cells are derived from distinct hematopoietic progenitors (122). Notably, neonatally-derived CD8+ T cells persist into adulthood, where they continue to play an important role in responding to pathogens due to their preferential differentiation into effectors that proliferate rapidly and produce cytokines (123, 124). In contrast, adult-derived CD8+ T cells in the same environment have a greater propensity to generate memory T cells (124). In uninfected mice, CD8+ T cells generated during the neonatal period tend to differentiate into “virtual memory” T cells (Tvm), expressing high levels of CD44, Eomes, and CD122, and they proliferate more rapidly and differentiate into short-lived effector cells following pathogen challenge, mirroring the neonatal CD8+ T cell pool (122, 124, 125). Consistent with findings in mice, human cord blood CD8+ T cells are also highly proliferative upon TCR stimulation (123), and undergo bystander activation, producing IFN-*γ*, TNFα, or IL-4, depending on the cytokine receptor (126, 127). Collectively, these findings suggest that the functional potential of neonatal naive CD8+ T cells is biased towards an innate-like effector phenotype.

Thus, perinatal CD4+ and CD8+ Tconv cells have distinct functional properties compared to their adult counterparts. Both cell-intrinsic changes in differentiation potential and priming by different microenvironmental cues result in CD4+ T cell responses biased towards a Th2 or Treg (see below) fate, which may protect the neonate from damaging inflammatory Th1 responses at a time when tissue homeostasis, including responses to commensal colonization, is being established. During this period, CD8+ T cells are biased to differentiate into short-lived effector cells, which can rapidly combat pathogenic threats at the expense of generating memory responses.

### Selection of Tconv Cells in the Perinatal Period

Previous studies suggest that negative selection is impaired in the perinatal compared to the adult thymus, resulting in decreased deletion of self-reactive thymocytes (69, 128, 129). In mice, susceptibility to experimental autoimmune encephalomyelitis (EAE) decreases between the perinatal and adult period, which correlates with increasing age-dependent negative selection of MBP (myelin basic protein) specific T cells (129). Also, in mice and humans, Tconv cells that mature in the perinatal thymus express higher levels of CD5 and Nur77 compared to those generated in adults (130, 131). CD5 and Nur77 levels correlate with TCR affinity for peptide-MHC (132, 133), suggesting that perinatal Tconv cells are more self-reactive compared to those generated in the adult thymus. While heightened self-reactivity could reflect impaired central tolerance, as discussed above, it is also possible that the threshold for positive selection is higher in the perinatal thymus, such that thymocytes with low-affinity TCRs are not efficiently positively selected, resulting in elevated CD5 levels on perinatal T cells (130). Regardless, higher TCR self-reactivity could enable T cells to respond quickly and effectively against multiple foreign antigens, despite the limited perinatal TCR repertoire (131, 134). Studies in mice have suggested another potential advantage of increased TCR self-reactivity: neonatal recent thymic emigrants enter a lymphopenic periphery, where CD5^hi^ T cells outcompete their CD5^lo^ counterparts for CD28 co-stimulation due to their increased affinity for self-pMHC. Their resultant increased sensitivity to the homeostatic cytokines IL-7 and IL-15 promote lymphopenia-induced proliferation to fill empty niches (135–139). Moreover, CD5^hi^ T cells have skewed effector potential in the periphery. CD5^hi^ CD4+ T cells are more prone to differentiate into Tregs (140), while CD5^hi^ CD8+ T cells express effector molecules such as Eomes, T-bet and Helios that promote T cell differentiation to an effector or virtual memory fate (134, 139). Thus, the increased self-reactivity of T cells selected in a neonatal thymus likely contributes to the characteristic rapid proliferation of neonatal CD4+ and CD8+ T cells in response to cytokine or antigen stimulation, as well as the altered differentiation potential biasing neonatal CD8+ T cells to become short-lived effector cells or Tvm, and CD4+ T cells to adopt a Treg fate. These studies emphasize that the strength of TCR signaling during thymic selection not only determines lineage fate decisions in the thymus but also influences peripheral effector T cell function.

### Declining Function of Tconv Cells During Aging

It is well established that T cell function declines with age, correlating with increased morbidity and mortality to infectious diseases and reduced responses to vaccination (2, 141, 142). While following a general pattern of age-associated decline, there is increased variability in immune responses between individuals with age, due in part to their lifetime histories of acute and persistent pathogen encounters (143, 144). As in the perinatal period, both cell-intrinsic and microenvironmental changes contribute to the age-associated decline in T cell function; however, the complex mechanisms that drive waning T cell immunity are not yet fully resolved (2).

CD4 T cells exhibit multiple functional changes with age. Reduced expression and production of IL-2 has been demonstrated following stimulation of mouse CD4+ T cells (145, 146). There is evidence for reduced IL-2 production in CD4+ T cells from elderly humans, but this decline has not been universally observed (2). CD4+ T cells from old mice were found to be functionally deficient in B cell activation, indicating reduced T follicular helper cell (Tfh) activity with age (147). Consistent with this notion, an age-associated decline in Tfh responses, along with diminished class-switched antibody levels were reported following viral infections in mice, non-human primates, and humans (143, 148). In addition, aging is associated with reduced IFN-*γ* ^+^ CD4 T cell responses to viral pathogens (143, 149). Age associated defects in CD4 activity could result from impaired T cell priming as aged CD4+ T cells exhibit cytoskeletal defects and poor immunologic synapse formation (150), reduced calcium flux upon TCR cross-linking (151), and defective metabolic reprogramming upon activation (152). These findings suggest cell-intrinsic defects impair CD4+ T cell responses in aged individuals. Cell-extrinsic defects also contribute to the decline in CD4 T cell function with age. For example, aged CD4+ T cells showed reduced homing to LNs following viral infections, despite the finding that expression levels of LN homing molecules (CCR7, CXCR4, PSGL1, and LFA1) were not decreased (148). However, levels of CCL21, which recruits naive T cells to LNs, were lower in draining LNs from old mice early after infection (148). Further support for cell-extrinsic defects was demonstrated by studies showing that the LN microenvironment deteriorates with age due, in part, to reduced IL-7 presentation and increased fibrosis (2, 153, 154),

Because T cells consist of multiple functionally distinct subsets, the defects in T cell function with age described above could reflect a change in subset composition and/or alterations in activity on a per-cell basis. Indeed, phenotypic analyses revealed an age-associated reduction in the proportion and numbers of naive T cells in humans, non-human primates, and mice (154–158). Recent comprehensive single-cell transcriptional profiling studies confirm the shift towards a higher frequency of effector-memory T cell subsets with age (159, 160). Notably, in mice, aging was associated with a stark increase in representation of cytotoxic CD4+ T cells, exhausted CD4+ T cells, and activated Treg (159). The shift to an increased frequency of these CD4+ T cell subsets correlated with elevated levels of cytokines associated with inflammaging, such as IL-27, IFN-β, and IL-6. Thus, the altered distribution of CD4+ T cell subsets likely has a profound impact on immune responses with age. However, such alterations do not fully account for age-related changes in T cell function. For example, antigen-inexperienced CD4+ recent thymic emigrants (RTEs) from old mice produce less IL-2 and proliferate poorly after *in vitro* stimulation compared to young RTEs (161). In addition, naive CD4+ T cells from older mice have a longer lifespan, reflecting increased *Bim* expression, but proliferate poorly after *in vitro* and *in vivo* stimulation (162, 163). Although profound functional deficiencies in naive human CD4+ T cells have not been reported, naïve CD4+ T cells from elderly humans exhibit reduced TCR signaling and expansion following *in vitro* stimulation due, at least in part, to the age-associated decline in miR-181a expression (164–166). Interestingly, naive polyclonal CD4+ T cells in aged mice are more self-reactive, as indicated by increased CD5 expression, display higher TCR affinity for foreign antigens, and are more promiscuous in antigen recognition. The increase in self-reactivity and promiscuity of the aged CD4+ T cell compartment implicate altered thresholds of CD4+ T cell selection in the thymus with age (155).

Defects in CD8+ T cell responses with age are well established. Early studies reported defective CD8+ T cell responses following primary and secondary influenza challenges (167). Additionally, CD8+ T cells have an age-associated decrease in their capacity to proliferate and produce effector molecules, such as IFN-*γ*, following *in vitro* stimulation or infection with viral or bacterial pathogens (168–175). In humans, the frequency of activated CD8+ T cells induced by yellow fever vaccination was significantly diminished with age (176), underscoring the potential impact of a declining CD8+ T cell compartment on vaccine-induced as well as on natural protection against pathogens (143).

Similar to CD4+ T cells, the overall decline in CD8+ T cell function with age could reflect changes in the proportions of functionally distinct CD8+ T cell subsets. Indeed, one of the most notable hallmarks of the aged immune system in humans and mice is a substantial decline in both the number and frequency of naive CD8+ T cells (143, 173, 177). At the same time, the CD8+ T cell pool becomes progressively enriched in clonally expanded, antigen-inexperienced CD8+ Tvm cells in mice and in humans (139, 174, 177–180). The homeostatic cytokine IL-15 is required for differentiation and function of Tvm cells (139), which in turn respond to IL-12 and IL-18 stimulation in a TCR-independent manner, resulting in secretion of IFN-*γ* (139, 174, 177–179). Notably, Tvm can provide antigen-independent bystander protection in bacterial infections, proliferating more rapidly than naive T cells, but differentiating preferentially into short-lived effector cells (181), strikingly reminiscent of perinatal Tconv cells. While Tvm cells can provide effective protection against pathogens in a bystander or TCR-dependent manner (139, 181), and increase in frequency with age, there is a seemingly incongruous age-associated decline in the overall response of CD8+ T cells to pathogen challenge. Previous studies partially resolved this conundrum by showing that aged Tvm in mice and humans have a reduced capacity to proliferate in response to TCR stimulation relative to young Tvm. The mouse Tvm response to homeostatic cytokines is sustained with age, but whether human Tvm have a similarly sustained response has not been tested (173, 174). Regardless of age, Tvm mount a monofunctional cytokine response to TCR stimulation, while naive CD8+ T cells produce multiple cytokines in response to mouse influenza infection (173, 174). These studies indicate that with age Tvm cells accumulate in the CD8+ compartment, respond poorly to TCR stimulation, and produce a less diverse cytokine response. In contrast, while naive CD8+ T cells retain a robust capacity to proliferate to TCR stimulation with age, they do not survive or proliferate well in response to the homeostatic cytokines IL-2 and IL-15, explaining the decreased proportion of naive CD8+ T cells relative to Tvm with age (173, 174). Transcriptional profiling revealed that Tvm cells that accumulate with age have a senescent signature, consistent with the lower frequency of cells that respond to TCR stimulation as well as the reduced burst size of individual responding cells (173). Together, these results partially explain why the composition and function of the CD8+ T cell compartment changes with age. Additional insights into the declining function of aged CD8+ T cells were revealed in a recent single cell transcriptional profiling study that identified a subset of CD8+ T cells that expresses and secretes granzyme K (GZMK) and accumulates with age in mice and humans (160). In contrast to Tvm, age-associated GZMK+ CD8+ T cells have a transcriptional profile and surface marker phenotype (PD-1+ Tox+) consistent with a state of terminal exhaustion (160). Strikingly, upon TCR stimulation, these cells secrete GZMK, which alone or in combination with IFN-*γ*, induces fibroblasts to secrete pro-inflammatory factors, such as IL-6 and CCL5. Thus, GZMK+ CD8 T cells may contribute to inflammaging. GZMK+ CD8+ T cells also express the integrin CD49d, reminiscent of a previously described clonally expanded CD49d+ CD8+ T cell subset in aged mice (182). These cells home to multiple tissues and fail to secrete granzyme B (GZMB) or IFN-*γ* upon TCR stimulation, further distinguishing them from Tvm (160). Notably, single-cell TCR repertoire analysis of human PBMCs revealed that the well-documented clonal restriction of the CD8+ T cell pool with age (177, 183–185) was due in part to clonal expansion of this novel GZMK+ CD8+ T cell subset, which was distinct from the clonally expanded GZMB-producing cells that are enriched for recognition of CMV or Epstein-Barr virus (EBV) (158, 186). Clonal expansion of Tvm with age has also been reported (174, 177, 185). Collectively, these studies reveal that aging is associated with a profound shift in the composition of CD8+ T cell subsets, resulting in reduced responses to newly encountered antigens and a shift towards a pro-inflammatory phenotype.

Age-associated changes in the composition of the CD8+ T cell compartment could reflect cell-intrinsic and/or extrinsic influences. Several lines of evidence indicate that the aged environment is a causative factor in the decline in CD8+ T cell functionality. When young naive CD8+ T or Tvm cells are transferred into an aged host, their proliferative potential declines (173). Similarly, an aged host environment induces young CD8+ T cells to adopt an exhausted phenotype, including upregulation of GZMK (160). Additionally, in heterochronic parabiosis experiments fewer young CD8+ T cells were recovered in old compared to young partners (187). Conversely, in each of these studies, the young environment did not restore function, cellularity or phenotype to old CD8+ T cells. Strikingly, the number of T cells declined in the lymph node of a young mouse when parabiosed to an old partner (187). Together, these data indicate that the old environment contains soluble factors that negatively impact CD8+ T cell cellularity and function. Additional cell-extrinsic influences that can diminish CD8+ T cell responses with age include ineffective antigen presentation by aged DCs (188, 189) and disrupted architecture of secondary lymphoid organs that could impair recruitment, maintenance or priming of CD8+ T cells (153, 154, 190, 191). Despite clear evidence that cell-extrinsic factors in the aged environment modulate CD8+ T cell responses, there is evidence that age-associated cell-intrinsic changes also contribute to diminished T cell responses with age. In addition to the declining responsiveness of aged Tvm to TCR stimulation (173, 174), another characteristic of Tvm cells that accumulate with age is their increased self-reactivity, as reflected by elevated expression of CD5 (139, 185). Furthermore, there is an apparent enrichment in naive T cells with higher CD5 levels in the CD8+ T cell repertoire with age (177, 192), and naive CD8+ T cells expressing higher levels of CD5 have an increased propensity to differentiate into Tvm cells. Together, these data indicate that the naive CD8+ T cell pool is more self-reactive with age. Further studies are needed to determine whether the increased self-reactivity of naive T cells is driven by age-associated cell-extrinsic changes in the thymic microenvironment that affect selection thresholds, peripheral maintenance of self-reactive T cells, and/or intrinsic transcriptional profiles of T cells that alter their capacity to respond to TCR signals.

### Changes in Negative Selection of Tconv Cells During Aging

Aging induces profound changes in the thymic microenvironment (see section *Changes In Thymic Apcs And Implications For Selection Throughout The Lifespan*), which could negatively affect central tolerance. For example, TRA expression decreases with age (193, 194), reflecting both a decline in the frequency of *Aire*+ mTECs and reduced *Aire* expression per mTEC (195, 196). Thus, thymocytes may not encounter the full spectrum of self-antigens responsible for central tolerance in an aged thymus, potentially contributing to the increased incidence of autoimmunity with age. Consistent with this possibility, *Aire* haploinsufficiency results in decreased negative selection and an increased incidence of diabetes (197). Also, in an inducible *Foxn1*-deletion model of accelerated thymic atrophy, TRA expression was reported to decline, and negative selection was impaired (198). In addition to age-associated changes in TECs, changes in thymic B cells could impact central tolerance during aging. The number and frequency of thymic B cells increase in old mice; however, their expression of *Aire* and TRAs diminishes with age (199–201). A decline in AIRE-dependent TRA expression is also observed in human thymic B cells (199). Despite the clear association between aging and thymic involution, and recognition that the thymic microenvironment is critical for establishing self-tolerance, surprisingly little is known about the impact of aging on central tolerance. Further investigations are needed to determine if central tolerance is altered during aging, to elucidate the underlying mechanisms, and to determine the impact on autoimmunity.

## Changes in Treg Function and Thymic Selection Throughout the Lifespan

### Function of Tregs in the Perinatal Period

The critical role of Tregs in suppressing damaging inflammatory immune responses in a broad range of tissues has been well documented [reviewed in (202)]. Immunodysregulation polyendocrinopathy enteropathy X-linked (IPEX) patients, in whom Treg lineage differentiation is impaired, develop severe gastrointestinal pathology, type-1 diabetes mellitus and severe skin inflammation, in addition to other autoimmune manifestations within the first few weeks to months after birth (203–206). Studies in mice have demonstrated that organ-specific Tregs play a crucial role in promoting peripheral tolerance in both lymphoid and non-lymphoid organs (207–209). Tregs control inflammatory T cell responses towards food antigens (210) and commensal microbiota in the gut (211), and intestinal Tregs have been shown to expand in response to microbial cues (211–214). Tregs also migrate to the hair follicles in the skin, where they are critical for tolerance to skin commensals (215, 216). Retinal antigen-specific Tregs in the eye control inflammation in experimental autoimmune uveitis and help resolve disease pathology (217, 218). Other experimental models of organ-specific diseases such as diabetes (219) and EAE (220) have reinforced the crucial role played by Tregs in suppressing autoimmune pathology.

Tregs control T cell responses through multiple mechanisms (221, 222). For example, expression of CTLA-4 on Tregs reduces the ability of DCs to stimulate T cell responses by masking the costimulatory molecules CD80 and CD86 (223). Tregs also express CD39 and CD73, which catalyze the release of adenosine into the extracellular milieu, thus inhibiting effector T cell proliferation (224). In addition, Tregs outcompete effector T cells for IL-2, inhibiting their proliferation (225), and Tregs produce suppressive cytokines like IL-10 and TGFβ (226, 227). Tregs can also suppress effector T-cell differentiation and induce apoptosis of Tconv cells (228, 229).

The importance of intrathymic Treg generation in the neonatal period is illustrated by an experiment performed nearly 50 years ago in which neonatal thymectomy in mice was shown to cause autoimmune pathology in the ovaries (230). Later studies showed that transfer of adult T cells, in particular CD25+ Tregs, prevented autoimmune destruction of ovaries in these mice, implying that a defect in neonatal thymic Treg generation failed to curb activation of autoreactive T cells (231, 232). Differentiation of Foxp3+ CD25+ Tregs in the mouse thymus lags behind that of Tconv cell development (233). In newborn mice, Tregs comprise only 0.09% of CD4SP thymocytes and do not reach adult levels (~4% of CD4SP thymocytes) until 21 days after birth (233). In contrast, CD25+ Tregs constitute ~6-8% of CD4SP thymocytes in humans by 14-17 gestational weeks (GW), and this frequency remains relatively constant after birth (234, 235). Perinatal expansion of the human Treg compartment is observed in the periphery, with a striking surge in the frequency and number of peripheral blood CD25+ Foxp3+ Tregs during the early neonatal period (7-8 days post birth) compared to those in cord blood or present at a later neonatal period (2-4 weeks after birth) (236). Additionally, compared to adult tissues, a higher frequency of Foxp3+ CD25+ Tregs is observed in human fetal as well as in several pediatric lymphoid and mucosal tissues, indicating their importance in early life (86, 237). Tregs in neonatal circulation display an activated phenotype, with a predominantly Foxp3^hi^ CTLA-4^hi^ CCR7^lo^ CD25+ phenotype (236). Similarly, an activated (CD69^hi^ GITR^hi^ CCR7^lo^ CTLA-4^hi^), memory (CD62L^lo^ CD45RO+) Treg phenotype was documented in fetal LN and cord blood (235, 237).

The high frequency of Tregs in the fetal and perinatal periods may be due to a higher propensity of fetal hematopoietic progenitors to differentiate into the Treg lineage. HSCs transplanted from human fetal liver or bone marrow into humanized mice give rise to a higher frequency of CD25+ Foxp3+ Tregs compared to HSCs from adult bone marrow (238). Additionally, studies in mice have demonstrated that perinatal CD4SP thymocytes are more prone to differentiate into Tregs upon TCR stimulation when compared to adult CD4SP thymocytes, both *in vitro* and *in vivo* (239, 240). Gene expression profiles of adult Tregs are more similar to fetal naive CD4+ T cells than to adult naive CD4+ T cells, indicating that fetal T-cells may be transcriptionally primed to be suppressive. Consistent with this finding, naive CD4+ T cells from human fetuses give rise to more Tregs than adult CD4+ T cells *in vitro* (236, 238). The increased Treg induction efficiency of perinatal progenitors could be a protective mechanism required to establish initial immune tolerance in multiple peripheral tissues, particularly in light of elevated Tconv self-reactivity in the perinatal period (see above). Supporting this theory, *Aire* expression in the perinatal period is necessary and sufficient to prevent autoimmunity in mice (241), and Treg ablation in perinates induces profound multiorgan autoimmunity characteristic of **Aire** deficiency (242). Together, these findings suggest that *Aire* expression in the perinatal thymus is essential for selecting perinatal Tregs that suppress multiorgan autoimmunity. Tregs are required for self-tolerance throughout life, as demonstrated by the autoimmunity that ensues following *Foxp3* elimination in adult mice (207). Notably perinatally-derived Tregs persist into adulthood, and relative to adult-derived Tregs, are uniquely capable of protecting against autoimmunity when transplanted into **Aire**-deficient mice. Perinatally-derived Tregs also express an activated gene signature and have an increased capacity to suppress Tconv cell proliferation *in vitro* relative to adult-derived Tregs (242). These mouse studies are consistent with human studies showing distinct gene expression patterns in fetal versus adult Tregs (238), as well as increased protein expression and suppressive activity of pediatric compared to adult Tregs (86). Taken together, these findings suggest that perinatally-derived Tregs persist into adulthood, where they suppress damaging autoreactive T-cell responses in multiple organs.

Many studies of Treg-mediated protection in tissues have been performed in adults, raising questions of whether neonatally derived Tregs play a critical role in these processes, and if so, what mechanisms underlie their suppressive activity. Some progress has been made towards answering these questions. Tregs generated in the neonatal thymus migrate to the skin in a CCR6-CCL20 dependent manner, where they are essential for establishing tolerance to newly colonizing commensal bacteria (216). Recent studies have also reported that a wave of neonatal thymus-derived Tregs migrates to the liver (243, 244). Interestingly, perinatal liver-resident Tregs are more suppressive than their splenic counterparts, and they are activated in a TCR-dependent manner in the liver microenvironment (243). Ablating these perinatal Tregs resulted in Th1-type inflammation and breakdown of lipid metabolism, highlighting their role in establishing liver homeostasis (243). Another study demonstrated that perinatal Tregs promote and maintain anergy of self-reactive PD-1+ CD44+ Tconv cells in the liver; notably development of these perinatal Tregs was Aire independent (244). These results contrast with the Aire-dependence of perinatal Tregs that confer protection against autoimmune infiltrates in *Aire*-deficient mice (242). Thus, the contribution of Aire to selection of perinatal thymic Tregs that suppress tissue-specific autoreactivity requires further investigation. Tolerance to commensals at mucosal barriers is established in the neonatal period and is mediated by peripherally-induced Tregs. In neonatal mice, the lung microbiota induce differentiation of a Helios negative Treg subset that suppresses Th2-like hyper-responsiveness to aeroallergens (245). Additionally, neonatal T cells encounter a wide variety of antigens derived from gut microbiota which induce Treg differentiation required for tolerance to gut commensals throughout life (246). Thus, thymus-derived and peripherally-induced Tregs are generated early in life and are critical for tissue-specific immune homeostasis in multiple organs.

### Selection of Tregs in the Perinatal Period

In mice and humans, Foxp3, the master transcriptional regulator of Treg lineage commitment and maintenance, is predominantly induced in self-reactive CD4SP thymocytes, although it can be detected as early as the DP stage (233–235). Perinatal Tregs express higher CD5 levels compared to adult Tregs, suggesting increased self-reactivity (130).Two distinct Treg populations that differ in their affinity towards self-antigens have been identified in adults (247). Triple^hi^ (PD-1^hi^ GITR^hi^ CD25^hi^) Tregs are more self-reactive, as indicated by higher Nur77 and CD5 levels, and are efficient at suppressing Tconv cell proliferation in lymphoid organs. In contrast, Triple^lo^ (PD-1^lo^ GITR^lo^ CD25^lo^) Tregs express less Nur77 and CD5, indicative of lower self-reactivity, and more effectively limit the induction of colitis by inducing peripheral Tregs in the gut (247). However, both Triple^hi^ and Triple^lo^ Tregs in the perinatal thymus express elevated CD5 levels relative to their adult counterparts (130). Taken together with the evidence that Tregs selected in the perinatal thymus are critical for suppressing autoimmunity at multiple tissue sites, higher CD5 expression by thymic perinatal Tregs suggests that the perinatal thymic environment may be specialized for selecting tissue-protective Tregs.

Recent studies support the possibility that self-antigen presentation differs in the perinatal versus adult thymus microenvironment, resulting in efficient Treg selection. A self-peptide derived from peptidyl arginine deaminase type IV (Padi4) was found to efficiently induce selection Treg only in the perinatal thymus (248). Interestingly, in adults, Padi4-specific thymocytes were subject to negative selection as early as the post-positive selection DP stage, whereas in perinates, negative selection was delayed until the CD4SP stage. Thus, in the adult thymus, Padi4-specific DP precursors were deleted before they could give rise to CD4SP cells or Tregs, likely underlying the switch from perinatal Treg induction to adult negative selection (248). The age-associated shift towards clonal deletion could reflect cell-intrinsic changes in signaling downstream of TCR stimulation in perinatal versus adult DP thymocytes and/or changes in the perinatal versus adult thymic microenvironment. In this regard, bone marrow chimera experiments revealed that expression of Padi4 by HAPCs induced negative selection in the adult thymus, but when Padi4 expression was restricted to radioresistant thymic stromal cells, Treg induction was restored in adults. These findings suggest that antigen presentation by adult HAPCs preferentially drives negative selection, as opposed to Treg induction. Conversely, unique properties of the thymic APC compartment in neonates may selectively promote thymic Treg induction over negative selection. The concept that age-associated changes in the thymic microenvironment play a role in the outcome of self-antigen recognition is supported by the lower expression of H2-DO relative to H2-DM in perinatal versus adult mTECs, which would increase the diversity of peptides presented in the perinatal thymus (242), thus altering the TCR repertoire during thymic selection. Collectively, these studies demonstrate that both negative selection and Treg induction differ in the perinatal versus adult thymus, yielding more autoreactive Treg and Tconv cells in the perinatal period. However, the mechanisms driving these age-dependent changes in selection thresholds and TCR specificities, including whether these differences are due to cell-intrinsic changes in T cell progenitors and/or cell-extrinsic factors in the thymic microenvironment, remain to be resolved.

### Changes in Treg Function During Aging

The prevalence of Tregs in the blood of adult mice and humans ranges from 5-10% of the CD4+ T cell compartment (249). The frequency of Tregs does not increase in mouse blood with age (250). In contrast, elevated frequencies of circulating Tregs have been reported in aging humans (251, 252). Furthermore, aging is associated with an increase in both the frequency and number of Tregs in mouse spleen and lymph nodes, but not in the lung, liver or peritoneum (160, 250, 251, 253, 254) In fact, a recent single-cell transcriptional profiling study confirmed that the frequency of Tregs increases in aging mouse spleens, but revealed that this increase was driven almost entirely by an emerging subset of activated Tregs (159). Taken together, these studies indicate that the abundance, distribution, and function of Tregs shift with age towards increasing immunosuppression.

Two single-cell transcriptomics reports show that with age, Tregs express elevated levels of genes associated with Treg activation and suppressive activity, including *Foxp3*, *S100a11*, *IL1r2*, *Pdcd1*, *Tigit*, *Lag3*, and *Batf* (159, 160). Moreover, expression of proteins that promote Treg suppressive activity, such as FOXP3, CD25, CTLA-4, and GITR, is maintained, and in some cases increased in aged Tregs (251, 252, 254, 255). A recent study reported that old activated Tregs are more suppressive than young Tregs (159), consistent with previous findings showing increased functional activity of Tregs with age (251). In contrast, other studies report that the *in vitro* suppressive capacity of Tregs does not differ between young and aged mice (256, 257) or humans (255). Nevertheless, whether due to increased frequency or increased suppressive capacity, it is likely that aged Tregs may impair T-cell mediated control of infection with age, thus contributing to pathology. In keeping with this concept, young mice are able to resolve primary *Leishmania major* infection, whereas aged mice experience increasing reactivation of lesions. However, Treg depletion in the aged mice increased cytokine production by effector T cells and decreased disease severity (251). In addition, CD4^+^ CD25^hi^ Tregs recovered from Alzheimer’s disease and Parkinson’s disease patients displayed increased suppressive activity *in vitro* when compared to young and control elderly donors, suggesting that Treg suppressive capacity is also associated with age-related neurodegeneration (252). Increasing Treg activity may also contribute to diminished anti-tumor responses with age. Whereas young mice were able to reject transplanted BM-185 tumor cells, aged mice succumbed, and their ability to reject tumors was restored by Treg depletion (254). Because there are multiple subsets of functionally distinct Tregs (258), some of the discrepancies above regarding alterations of Treg functionality with age may reflect changes in the composition of Treg subsets, which could be impacted by organ sites and the assays chosen to measure Treg suppressive activity. Consistent with this possibility, Tregs were found to be more abundant in the oral mucosa of aged mice and humans, although, counterintuitively, inflammation associated with *Candida albicans* infection was exacerbated despite pathogen control (259). Notably, an age-related shift in favor of IFN-*γ*-producing relative to IL-17-producing Tregs and Tconv cells was associated with decreased IL-1β and increased IL-6 levels in the mucosa. IL-1R1 deficiency decreased induction of IL-17-producing Tregs after *Candida albicans* infection, whereas there was a relative increase in IFN-*γ*-producing Tregs, which required IL-6 for their expansion (259). In a mouse model of autoimmune colitis, aged Tregs could suppress IFN-*γ*+ Th1 cells, but not IL-17+ Th17 cells (260). Restraint of Th17 cells requires STAT3 activation in Tregs (261), and aged Tregs do not activate STAT3 in response to inflammatory IL-6 to the same extent as young Tregs (260). Collectively, these studies demonstrate that age-associated changes in the relative abundance of different cytokines, as well as the responsiveness of aged Tregs to cytokine stimulation, can alter Treg subset differentiation and thus, the ability to suppress inflammatory T cell responses to self-antigens, pathogens, and commensals in a tissue-specific manner. While changes in cytokine levels would contribute to extrinsic alterations in Treg differentiation and function with age, changes in the ability of aged Treg to respond to cytokines suggest that age-associated cell-intrinsic changes affect Treg function.

### Selection of Treg With Age

The absolute number of Tregs in the thymus decreases with age, reflecting the reduction in cellularity that accompanies age-associated thymic involution (250, 253). Although the frequency of FOXP3+ cells does not change with age (250, 253), initial studies did not distinguish between thymic Tregs generated in the aged thymus versus those that had recirculated into the thymus from the periphery. Subsequent studies using RAG2p-GFP mice revealed that the frequency of newly generated Tregs declines rapidly with age, while the proportion of recirculating Tregs increases (262, 263). Moreover, mature Tregs inhibit *de novo* generation of Tregs in fetal thymic organ cultures, suggesting that recirculating Tregs reduce selection of new Tregs in the aged thymus, perhaps by sequestering IL-2, a limiting cytokine required for Treg induction (264). In this regard, Treg generation was increased in the presence of exogenously administered IL-2 (262). These studies suggest that thymic Treg induction is reduced with age. In contrast, Treg selection was favored over clonal deletion in an inducible *Foxn1*-deletion model of accelerated thymic involution, in which TECs are precipitously depleted (198). Thus, it remains to be resolved whether Treg generation is generally reduced in an aged thymus, or is actually increased under some conditions, such as limited self-antigen availability.

Given that the number of Tregs in the periphery does not decline with age, and in fact increases in some organs (see above), the decline in thymic output of newly generated Tregs during age-associated thymic involution must be compensated for in the periphery either by increased proliferation/survival of extant Tregs or increased Treg induction. Naïve CD4^+^ T cells from old mice have a diminished ability to differentiate into Tregs *in vitro* and *in vivo* (188, 265). However, aged Tregs have a survival advantage relative to young Tregs due to lower expression of the pro-apoptotic factor *Bim* (253, 266). It is important to note that there are multiple subsets of peripheral Tregs (267–271), such that age-associated increased Treg survival could reflect an increased proportion of a long-lived subset. In keeping with this possibility, CD25^lo^ Tregs accumulate with age in the periphery (256, 266). CD25^lo^ Tregs express lower levels of *Bim* than CD25^hi^ Tregs, even though *Bim* levels decline in CD25^hi^ Treg with age (266). Notably, IL-2 is critical for homeostasis of CD25^hi^ Tregs and IL-2 levels decline with age, whereas the CD25^lo^ subset requires IL-15 for survival (266). Thus, altered access to homeostatic cytokines could impact the relative proportions of different Treg subsets with age, which would be in keeping with both the observed decline in circulating IL-2 and the age-associated deterioration of a supportive T-cell microenvironment in secondary lymphoid organs (2), especially given that autoreactive CD4+ T cells in secondary lymphoid organs are an important source of IL-2 for Tregs (272). Thus, there are age-related consequences for Treg selection, induction, and maintenance in the thymus and in the periphery.

## Changes in Thymic APCs and Implications for Selection Throughout the Lifespan

### Changes in TECs Across the Lifespan

The composition and function of TEC subsets undergo major changes throughout the lifespan, and there is mounting evidence that the dynamic nature of the TEC compartment is a critical determinant of age-associated alterations in the immune response. As previously discussed, mTECs play a critical role in establishing and maintaining central tolerance. Not only are mTECs uniquely capable of expressing and presenting Aire-dependent and Aire-independent TRAs (38, 43), but they also transfer TRAs to DCs for subsequent cross-presentation to thymocytes (53, 62, 63). In addition, mTECs produce chemokines such as XCL1, CCL19, and CCL21 that promote DC medullary recruitment and localization (70, 273, 274). Moreover, in response to Toll-like receptor (TLR) signaling, mTECs secrete chemokines that recruit CD14+ monocyte-derived DCs into the medulla to promote Treg generation (275). Thus, mTECs play multifunctional and essential roles in negative selection and Treg generation.

The mTEC compartment in both humans and mice is phenotypically and functionally heterogeneous. Initially, mTECs were classified into two major subsets, namely an immature MHCII^lo^ CD80^lo^ AIRE^-^ subset (mTEC^lo^) and a functionally mature MHCII^hi^ CD80^hi^ AIRE^+^ subset (mTEC^hi^). There is long-standing evidence that the mTEC^lo^ compartment contains progenitor cells that generate mTEC^hi^ progeny (276–278). However, it is now evident that the mTEC^lo^ population is highly diverse and contains multiple functionally and developmentally distinct subsets that have been identified in investigations using flow cytometric as well as lineage tracing and transcriptomic analyses of mouse and human mTECs. For example, a subset of mTEC^lo^ cells expresses CCL21, indicating their functional importance in recruiting positively selected thymocytes into the medulla (279–283). Interestingly, despite the initial association of an mTEC^lo^ phenotype with an immature stage of differentiation, the mTEC^lo^ subset also contains mature cells that have downregulated Aire and MHCII expression (44, 279, 280, 282). Studies employing single-cell RNA sequencing (scRNAseq) analyses have shown that post-Aire mTECs include a unique population of thymic tuft cells, which are sensory epithelial cells similar to those present in the intestine and other mucosal sites (279, 280, 282). It has been suggested that tuft cells play a role in central tolerance, as the abundance of Foxp3^lo^ Treg precursors decreases in tuft cell-deficient mice (284). Hassall’s corpuscles (HCs) are another cell type in the heterogeneous post-Aire mTEC^lo^ subset. HCs form distinctive concentric structures of flattened epithelial cells that are prominent in the human thymus medulla, and small clusters of TECs that may be analogous are found in mouse medullary regions. Transcriptional profiling studies have identified genes that are highly expressed by both HCs and terminally differentiated keratinocytes (279, 282). Moreover, HCs resemble keratinocytes in that both cell types produce proteins found in terminally differentiated epithelial cells such as keratin 10, involucrin, filaggrin and TSLP (44, 285–287). It has been suggested that HCs play a role in regulating central tolerance as TSLP produced by human HCs activates DCs to express co-stimulatory molecules that enhance Treg induction (78). A recent study in which scRNAseq analysis was performed on index-sorted TECs identified a novel TEC subtype, referred to as intertypical, which has both mTEC and cTEC characteristics (193). Thus, studies to date have shown that the mTEC compartment is highly diverse, consisting of multiple subsets whose phenotypic and functional characteristics, as well as lineage relationships have not yet been fully deciphered. Nevertheless, there is mounting evidence that various mTEC subsets significantly impact the establishment and/or maintenance of central tolerance.

The TEC compartment is highly dynamic during the perinatal to adult transition. TEC numbers expand exponentially during mouse fetal thymus development, and TEC cellularity continues to increase in the perinatal period prior to temporarily leveling off in young adults (288, 289). In parallel, there is a higher frequency of proliferating TECs in the perinatal compared to adult thymus (279, 288, 290). Remodeling of the TEC compartment during the perinatal period in mice is reflected by an increase in the percentage of mTECs and a corresponding decline in the percentage of cTECs (289, 291). Interestingly, functional blockade of vascular endothelial growth factor (VEGF) receptors in neonatal mice inhibits perinatal thymus expansion and accelerates the shifted mTEC to cTEC ratio despite the lack of VEGF receptors on thymocytes and TECs (292). These effects were independent of changes in the vasculature; however, VEGF inhibition altered expression of genes regulating cellular adhesion, migration, adipogenesis and inflammation in CD140a+ mesenchymal cells suggesting that VEGF-mediated effects on mesenchymal stromal cells influences changes in the TEC compartment during the perinatal period (293). The relative increase in mTECs during the perinatal to adult transition was found not only by flow cytometric analysis, but also by microscopic analysis of histological sections (289) showing that this change is not merely an artifact of the enzymatic digestion procedure required to obtain single thymus cell suspensions. This is a matter of concern because enzymatic disaggregation results in suboptimal recovery of cTECs, particularly those present in cage-like structures, referred to as thymic nurse cells, which encompass DP thymocytes (288, 289, 294). An increase in the frequency of mTECs relative to cTECs was also demonstrated by single cell transcriptional profiling of neonatal versus adult human thymuses (295). Furthermore, a recent scRNAseq analysis revealed the presence of a unique cTEC subset in the perinatal mouse thymus that rapidly declined and was replaced by mature cTECs in the adult thymus (193). The composition of the mTEC compartment also changes as perinates transition into adulthood. For example, few tuft cells are present in the neonatal mouse thymus, but their numbers increase substantially in adults (279, 281). Similarly, HCs become more abundant after the perinatal to adult transition (296). Taken together, these studies show that the network of TEC subsets undergoes extensive remodeling during the perinatal to adult transition.

TECs also undergo dynamic changes at the opposite end of the age spectrum as the thymus undergoes involution, a general feature of vertebrate aging. Thymus involution is characterized by progressive organ atrophy, reduced T cell output, disruption of thymus architecture and collapse of the TEC compartment (193, 297–300). Although both thymocyte and TEC cellularity decline as the thymus undergoes involution (161, 194, 288, 289, 291, 297), TEC depletion is a primary factor driving thymus involution. FOXN1, a transcription factor required for TEC development and maintenance, declines with age in mice and humans (291, 301–303). Genetic models in which *Foxn1* expression is upregulated in TECs prior to or after thymus involution can attenuate or reverse this process (195, 304), whereas downregulation of *Foxn1* results in early degeneration of the TEC compartment and premature involution (291). Furthermore, thymus involution can be prevented by expressing either a *Cyclin D1* or *c-myc* transgene in TECs, or by deleting *Retinoblastoma* family genes, all of which result in a continuous thymus growth phenotype despite the fact that thymocytes are not genetically manipulated (290, 305, 306). Furthermore, heterochronic parabiosis experiments showed that migration of thymus-seeding hematopoietic cells from a young partner into the thymus of an aged partner failed to restore cellularity of the old, involuted thymus (307). Collectively, these investigations indicate that degradation of the TEC compartment is a major factor contributing to thymus involution, a finding that is not surprising given that TEC-derived signals are indispensable for T cell differentiation and selection.

Although thymus involution is generally thought to result in a progressive decline in the number of both cTEC and mTEC compartments, this view was challenged by a recent investigation showing that the extensive cytoplasmic projections characteristic of cTECs contract during involution (308). Based on these findings, it was suggested that changes in cTEC morphology, rather than cellular depletion, are responsible for the apparent reduction in cTEC cellularity and associated cortical thinning (308). In contrast, morphological changes in mTECs were not observed during thymus involution consistent with an age-associated decline in the number of mTECs. In addition, changes in mTEC gene expression patterns, including increased expression of inflammatory pathway genes (193, 309) occur during thymus involution. The mechanisms responsible for transcriptional changes may reflect altered mTEC subset composition and/or intrinsic alterations in transcriptional regulation (193, 195, 196, 309). With regard to the former possibility, a recent study combining scRNAseq and lineage tracing approaches demonstrated marked changes in TEC subset composition with age (193). Taken together, these studies show that remodeling of the TEC compartment is a characteristic and progressive feature of age-related thymus involution.

Depletion of the mTEC compartment during aging, particularly the decline in mature mTECs that express Aire-dependent TRAs (195, 196, 288, 291), is likely to compromise central tolerance and result in increased export of self-reactive T cells. Indeed, a decline in expression of Aire-regulated as well as Aire-independent TRAs has been associated with age-related thymus involution (193, 194). Interestingly, however, neither the expression of *Aire* nor *Fezf2* (required for expression of *Aire*-independent TRAs) was altered in TECs obtained from aged, involuted thymuses suggesting that TRA expression depends on additional, as yet undefined, factors (193, 194). Collectively, these data suggest that the decline in mTEC cellularity, changes in mTEC subset composition and altered transcriptional signatures of mTEC subsets are features of thymus involution that may affect central tolerance and contribute to the age-associated increase in autoimmunity (3, 310).

### Changes in HAPCs Across the Lifespan

While mTECs present self-antigens directly to thymocytes to mediate central tolerance, DCs also cross-present mTEC-derived TRAs to induce negative selection and Treg generation (48, 53, 54, 62, 66, 71, 73, 74). Changes have been documented in the composition of thymic DCs during the perinatal to adult transition. Some studies indicate that CD8α^+^ cDC1s increase in the thymus of adult relative to fetal and neonatal mice (242, 311). However, another study showed that CD8α^+^ cDC1s decrease during the transition from neonate to adult, whereas Sirpα+ cDC2s and pDCs increase with age in the thymus (69). Adult cDC2s express higher levels of genes associated with antigen processing and presentation and are more efficient at MHCII-dependent antigen processing and presentation to T cells compared to newborn cDC2s. Interestingly, this study indicates that the efficiency of negative selection is diminished in perinatal mice, compared to adults, correlating with a lower frequency of cDC2 cells (69). In the human thymus, XCR1+ cDC1s increase in the second trimester of pregnancy and decline postnatally with increasing age (295), consistent with the trend reported in mice (69). Thus, while multiple studies have established that the DC compartment changes with age, the cellular composition and molecular alterations remain to be elucidated.

Since mTECs influence thymic DC localization and composition, mTEC-DC crosstalk likely plays an important role in mediating central tolerance. For instance, in the adult thymus, mTECs express CCL2, XCL1, JAG1, CCL19, and CCL21, which could promote recruitment, localization and/or maturation of thymic DCs (30, 33, 34, 61, 274, 295, 312–314). CCR7 is expressed not only by thymocytes, but also by some thymic DCs, indicating that expression of CCL19 and CCL21 by mTECs could recruit not only post-positive selection thymocytes to the medulla, but also CCR7^+^ DCs. CCR7 is upregulated on thymic DCs by interactions with autoreactive thymocytes in a CD40-CD40L dependent manner (65). CCR7+ DCs in human and mouse fetal and postnatal thymuses express high levels of MHCII and costimulatory molecules, suggesting they may have an increased capacity to present self-antigens to medullary thymocytes (64, 65, 295). Indeed, compared to CCR7- cDC1s, CCR7+ cDC1s are more efficient at acquiring and presenting *Aire*-dependent TRAs to CD8SP cells (64) and CCR7+ cDC1s have also been implicated in playing a central role in presenting mTEC-derived antigens to promote Treg selection (48, 66). We found that CCR7 deficiency results in increased apoptosis of MHCII^hi^ cDC1s and reduced antigen transfer from mTECs, with a concomitant increase in cDC2 and, surprisingly, Treg induction (70). Thus, production of CCR7 ligands by mTECs alters the composition of the thymic DC compartment, with downstream consequences for central tolerance. Taken together with the age-associated changes in DCs and mTECs, these findings suggest that altered mTEC-DC crosstalk in the perinatal period likely impacts central tolerance induction.

While the perinatal thymic HAPC compartment has slowly garnered interest over the last two decades, there is relatively less information regarding the impact of age-associated thymic involution on thymic HAPCs. The numbers and proportions of CD8α+ cDC1s and pDCs decrease gradually with age in the mouse thymus, while migratory Sirpα^+^ cDC2s remain constant in number, thus comprising an increased proportion of the thymic DC compartment (315). Though reduced in number, DCs in aged mice express similar levels of the activation markers CD40, CD80, CD86, and MHCII compared to young DCs, suggesting they are functionally intact (315). In humans, the number of total thymic DCs declines in proportion to the overall decrease in cellularity of the thymus with age (316). Similar to the mouse thymus, the proportion of DCs expressing MHCII, CD80, and CD86 was not altered with age, though expression of CD40 was diminished with age (316). Our transcriptional profiling analysis showed that murine thymic cDC1s and cDC2s express an increasingly proinflammatory gene signature with age, including expression of *Il1a, Il6, Tnf* and *Il18* (309). These aging DCs could potentially contribute to the age-associated inflammation observed in the thymus (317) and alter central tolerance. As previously mentioned, B cells mediate negative selection (81–83) and Treg induction in the thymus (318), underscoring their crucial contribution to central tolerance. Although the frequency of thymic B cells increases with age, there is a dramatic age-associated decline in Aire and Aire-dependent TRA expression in mouse and human thymic B cells (199), which could impair negative selection and Treg induction. These studies suggest that age-associated changes in HAPC subset composition and/or gene expression may impact central tolerance, highlighting the need for more comprehensive studies to determine how age-associated changes in thymic HAPCs influence negative selection, Treg induction, and the incidence of autoimmunity.

## Conclusions

The cellular composition and transcriptional profiles of TECs and HAPCs undergo profound changes throughout life, suggesting that a concomitant change in central tolerance likely occurs. Indeed, multiple lines of evidence presented above suggest that the perinatal thymic microenvironment is inefficient at inducing negative selection of Tconv cells, but is biased towards selection of auto reactive Tregs that are critical for suppressing autoimmunity in various tissues throughout life. On the other end of the aging spectrum, evidence suggests that the involuting thymus becomes inefficient at supporting both negative selection and Treg induction. The impact of age on the ability of the thymus to support both arms of central tolerance warrants further investigation. Studies to date raise several important issues. For example, while negative selection may be inefficient in both the perinatal and aged thymus, only the aged thymus appears to be impaired in supporting Treg generation, invoking a possible link with the age-associated increase in incidence of autoimmune disorders. Moreover, given that age-related changes in peripheral T cell differentiation and maintenance can impact the outcome of T cell responses to self and foreign antigens, it will be important to distinguish thymic from peripheral contributions with regard to changes in Tconv and Treg cells as a function of age.

Given that expression levels of CD5 on T cells are set during positive selection in the thymus, and that the level of CD5 on Tconv and Treg cells correlates with altered activity in the periphery, elevated CD5 levels on Tconv and/or Treg cells in the neonatal and aged periods indicate that age-associated changes in thymic selection impact peripheral T cell responses. However, changes in thymic selection could reflect either an altered capacity of thymic APCs to induce selection or cell-intrinsic changes in the differentiation potential of hematopoietic progenitors that seed and differentiate within the thymus. For example, the bias of neonatal CD8 T cells towards short-lived effectors reflects altered functional potential of neonatal versus adult hematopoietic cells (122). Whether preferential differentiation of CD4SP cells into Th2 effectors and Tregs in perinates reflects changes in T cell differentiation in the periphery and/or altered thymic selection, due to either an altered microenvironment or changes in hematopoeitic progenitors, requires further investigation. Furthermore, the mechanisms underlying thymic selection of T cells with altered self-reactivity have not been firmly established. Elucidating altered functions of Tconv and Treg cells with age will enhance understanding of how the immune system responds to pathogens throughout life without invoking autoimmunity. Furthermore, determining the mechanisms underlying altered thymic selection and peripheral maintenance of functionally distinct T cell subests will inform future strategies for enhancing T cell mediated immune protection and suppressing autoimmunity.

While studies to date have identified unique aspects of immune responses at both extremes of the age spectrum, and suggested that central tolerance is subject to age-related restrictions, a number of questions remain unanswered. Some of these unresolved questions include:

1. What are the precise roles of diverse mTEC and HAPC subsets in selection across the lifespan?
2. Is the bias of perinatal Tconv towards Th2 and short-lived effector cells due to altered thymic selection? Although this was found not to be the case for short lived CD8 effector cells (85), this question remains open for CD4^+^ T cells.
3. Do Aire-dependent versus Aire-independent Treg subsets induce anergy in Tconv cells in a tissue-specific manner?
4. What cellular subsets and molecular mechanisms account for the propensity of the perinatal thymic microenvironment to select CD5^hi^ Tregs?
5. Are the activated Tregs that accumulate with age and have heightened suppressive ability (159) essential for maintaining tolerance with age? A related issue is whether Tregs generated in adulthood contribute to tolerance.

## Author Contributions

JS and JL reviewed the literature. JS, JL, NS and LH prepared figures and legends, and commented on manuscript drafts. LE and ER revised the manuscript. All authors contributed to the article and approved the submitted version.

## Funding

Work in the laboratories of LH, LE and ER is supported by National Institutes of Health grants P01-AG052359 and P01- AI139449.

## Conflict of Interest

The authors declare that the research was conducted in the absence of any commercial or financial relationships that could be construed as a potential conflict of interest.
